# The Neuronal Transcription Factor Creb3l1 Potential Upregulates Ntrk2 in the Hypertensive Microenvironment to Promote Vascular Smooth Muscle Cell-Neuron Interaction and Prevent Neurons from Ferroptosis: A Bioinformatic Research of scRNA-seq Data

**DOI:** 10.1155/2022/8339759

**Published:** 2022-02-10

**Authors:** Xiaoyu Zhao, Jie Yang, Chuanhua Yang

**Affiliations:** ^1^Institute of Tradition Chinese Medicine, Shandong University of Traditional Chinese Medicine, China; ^2^Department of Cardiology, The Affiliated Hospital of Shandong University of Traditional Chinese Medicine, China

## Abstract

**Background:**

There is still a lack of knowledge regarding the association between hypertension and ferroptosis. A single-cell approach was used to study the changes in neuropeptide expression as they might contribute to the mechanisms leading to ferroptosis in a hypertensive microenvironment.

**Methods:**

We analyzed 11798 cells from the SHR group and 12589 cells from the WKY group of mouse arterial cells. CellPhoneDB was used for cell communication analysis, and the SCENIC method was used to identify key transcription factors in neurons. The correlation between Ntrk2 and ferroptosis-related genes was further analyzed and validated via quantitative polymerase chain reaction.

**Results:**

The arterial cells were clustered into six cell types. Ligand-receptor analysis suggested that Ngf, Ntf3, Cxcr4, and Ntrk2 were key neuropeptide-related genes involved in the communication between vascular smooth muscle cells and neural cells. In the hypertensive microenvironment, the neuronal transcription factor Creb3l1 appears to play a key role in the upregulation of Ntrk2 to promote the interaction between neurons and vascular smooth muscle cells. An association between Ntrk2 and the ferroptosis death inhibitor Gpx4 was suggested. RT-qPCR experiments confirmed that Ntrk2 downregulation in neural cells was followed by downregulated expression of Gpx4.

**Conclusions:**

Creb3l1, a key transcription factor in vascular neurons, may upregulate Ntrk2 to promote vascular smooth muscle cell-neuron interaction and thereby potentially prevent ferroptosis in neurons.

## 1. Introduction

Hypertension is an important risk factor for many cardiovascular and cerebrovascular diseases. In hypertension, a combination of platelet activation due to blood flow stagnation, endothelial cell damage, and abnormal shear stress causes coagulation in and damage to the arteries, which stimulates the bone marrow to strengthen its ability to proliferate platelets. This stimulation persists for a long time, maintaining the platelet count at high levels in patients with hypertension. This synergistic effect of hypertension increases the risk of cardiovascular disease [1, 2].

The prevalence of stroke has been increasing year over year. A large body of literature has previously reported that hypertension is significantly more likely to be complicated by thromboembolic diseases such as myocardial infarction and stroke than by hemorrhagic disease. This trend may be related to the presence of a prethrombotic state (PTS) in hypertension. The PTS (prethrombotic state) is a pathological process in which the body's hemostatic and coagulation functions are rendered dysfunctional caused by various factors [3]. The pressure load generated by a long-term increase in blood pressure causes direct damage to the vascular wall and endothelium, resulting in the activation of the coagulation and fibrinolytic system, which ultimately induces the formation of a PTS [4]. Hypertensive disease-related PTS is inextricably linked to hypertensive target organ damage—especially the heart and brain—and long-term prognosis.

The key to the occurrence of thrombosis is vascular endothelial damage, and its formation is a complex process. The vast majority of clinical or basic research now suggests that thrombosis occurs as a result of a combination of factors that include damage to the vascular endothelium, activation of procoagulant factors, diminished anticoagulant function, dysregulation of the fibrinolytic system, and altered blood rheology. The modified “damage response” theory proposed by Ross states that endothelial damage, especially endothelial dysfunction, was the initiating link of thrombosis formation. Following this, changes in endothelial permeability, adhesion, and blood coagulation would begin a series of chain reactions, supported by the release of large amounts of cytokines and growth factors secondary to endothelial damage. This cascade can, in theory, be prevented by protecting the endothelium [5, 6].

Neuropeptides are associated with endothelial damage. They are also known to be regulated by hypertension, and several neuropeptides are closely linked to the cardiovascular system. PACAP, for example, is a potent vasodilator that can relax the blood vessels of many organs, such as the brain, eyes, lungs, kidneys, skin, and ovaries. In addition, it also has the effect of lowering blood pressure, reducing vascular resistance, and increasing local blood flow [7]. It has additionally been shown that hypertension can cause endothelial damage via neuropeptides but the exact mechanism is still unknown.

There is still a lack of knowledge regarding the association between hypertension and ferroptosis. Ferroptosis, a concept first introduced by Dixon in 2012, is an iron-dependent, nonapoptotic form of cell death characterized by the accumulation of lipid reactive oxygen species (ROS) [8]. Lipid peroxides, particularly Fe2+, oxidize lipids in a Fenton reaction, generating large amounts of ROS and promoting ferroptosis. Recent studies have revealed that aberrant expression of some neuropeptides may be critical in the induction of ferroptosis in cells [9, 10]. Dysfunction of vascular neurons is an important feature of the hypertensive microenvironment [9]. Recent studies have identified hypertension as a potential cause of ferroptosis in cardiovascular neurons [11]. We speculate that the hypertensive microenvironment may contribute to the development of vascular neuronal ferroptosis, which may be a key cause of vascular endothelial injury in the hypertensive microenvironment.

With the establishment and development of single-cell genome sequencing, the understanding of diseases and the analysis of genomic features have entered the single-cell level [12, 13]. Single-cell sequencing technology is a powerful tool for studying cellular heterogeneity since it can reveal the gene structure and expression status of individual cells [14–16]. This technology has been used to unravel the cellular composition, immune cell status, cellular phenotype transformation processes, and complex intercellular mechanisms, laying the theoretical foundation for the cause, development, and treatment of hypertension [17].

This study aims at investigating, using a single-cell approach, the changes in neuropeptide expression and the mechanisms leading to endothelial damage in the hypertensive environment. In doing so, we hope to reveal the principles of hypertension that lead to thrombosis and arterial obstruction.

## 2. Materials and Methods

### 2.1. Data Collection

Data of single arterial vascular cells were obtained from the GSE149777 dataset of the GEO database. The dataset contains spontaneously hypertensive rats (SHR) and healthy control Wistar (WKY) rats aged 16–17 weeks [18]. The cellular heterogeneity in the mesenteric artery (MA) and aortic artery (AA) was reported by this database. A total of 11798 cells from the SHR group and 12589 cells from the WKY group were reported. Neuropeptide-related genes were based on the search results from GeneCards (https://www.genecards.org/) database using the keyword “Neuropeptides” and a cutoff score of 2. Ferroptosis-related genes were also obtained as described in previous studies [19–21].

### 2.2. Construction of a Cell Subpopulation Distribution Map

The percentage of mitochondria in the cells was analyzed. The Seurat algorithm package for R was used for further regression analysis of the expression data using log normalization to correct for potential intersample sequencing batch effects. The dispersion and expression values of genes were calculated to obtain high-variable genes (HVGs). Based on principal component analysis (PCA) of the scale data of the first 2000 HVGs, tSNE downscaling analysis was performed using the first 11 principal components to visualize and map the tSNE localization of single cells and key molecules for all samples. Normalized cell matrices were obtained by Seurat normalization to calculate cell communication significance and significance means. The FindMarkers function and Wilcoxon rank-sum test were used to identify differentially expressed genes between samples using the settings logFC > 0.25, *p* < 0.05, and minimum pct > 0.1. The study of Cheng et al. was referenced for cell annotation [18].

### 2.3. Analysis of Intercellular Communication

Ligand-receptor analysis is a good way to understand the state of interaction between different types of cells in the microenvironment [15, 22, 23]. To systematically analyze the cell-cell communication network, we used CellPhoneDB, a public knowledge base of ligands, receptors, and their interactions, for the annotation of membrane proteins, secreted proteins, and peripheral proteins clustered at different time points and for the calculation of their significance means and cell communication significance [24].

### 2.4. Identification of Key Transcription Factors in Neurons

Single-cell regulatory network inference and clustering (SCENIC) is a method to compute single-cell transcriptome data for gene regulatory network (GRN) reconstruction and cell state identification based on gene regulatory network and motif analysis [25–27]. A GRN consists of the TF and cofactor with its regulatory target gene, which determines the transcriptional state of the cell in a given state. The SCENIC process consists of three steps: (1) use of GENIE3 or GRNBost to infer coexpression modules between transcription factors and candidate target genes based on coexpression, (2) cis-regulatory motif analysis and TF-motif enrichment analysis performed for each coexpression module using RcisTarget to identify direct targets, and (3) scoring each cell for each regulon activity using the AUCell algorithm. The result generates a binary regulon activity matrix that may be used to identify key transcription factors in neurons.

### 2.5. Cell Culture

Immortalized lines of transmissible mouse hippocampal neuronal HT22 cells were cultured in DMEM complete medium containing 10% fetal bovine serum at 37°C in a volume fraction 5% CO2 cell culture incubator and digested with 2.5 g/L trypsin when confluence was between 70 and 80%. Subsequently, about 5000 cells per well were inoculated into the wells of 96-well plates for subsequent experiments.

### 2.6. Reverse Transcription Quantitative Polymerase Chain Reaction (RT-qPCR)

DNAMAN software was used to calculate the typical sequence of the CDS region of different transcripts of the same gene obtained from the PubMed website and designed the upstream and downstream primers that could span the intron of the gene using Oligo software. Finally, the specificity of the primers was verified by the BLAST function on the PubMed website and OligoCalc was used to check online whether the primers formed a hairpin structure and whether the 3′ ends formed a complementary structure. SYBR Green and upstream and downstream primers were mixed in clean Eppendorf tubes. The total amount of SYBR Green and upstream and downstream primers required was calculated based on the number of wells spiked for each indicator. After centrifugation, the samples were spiked on ice. After spiking, the samples were again centrifuged at 2000 rpm for 1 minute at 4 degrees. The program setting on the qPCR machine was adjusted to read the cycle threshold value of each well.

### 2.7. Data Analysis

Statistical and visual analyses were performed using R software (version 3.6.0) and GraphPad 6.0. The significance levels of the results of gene ontology (GO) and Kyoto Encyclopedia of Genes and Genomes (KEGG) enrichment analysis were calculated using Fisher's exact test, and the significance threshold was defined as *p* < 0.05. Samples with normal distribution were analyzed using a *t*-test between two independent samples; if they were nonnormally distributed, nonparametric tests were used. The results were expressed as the mean ± standard deviation (mean ± SD). Correlation analysis was done using Pearson's analysis, with statistical significance defined as *p* < 0.05.

## 3. Results

### 3.1. Determination of Each Subpopulation of Mouse Arterial Cells

**Figure 1:**
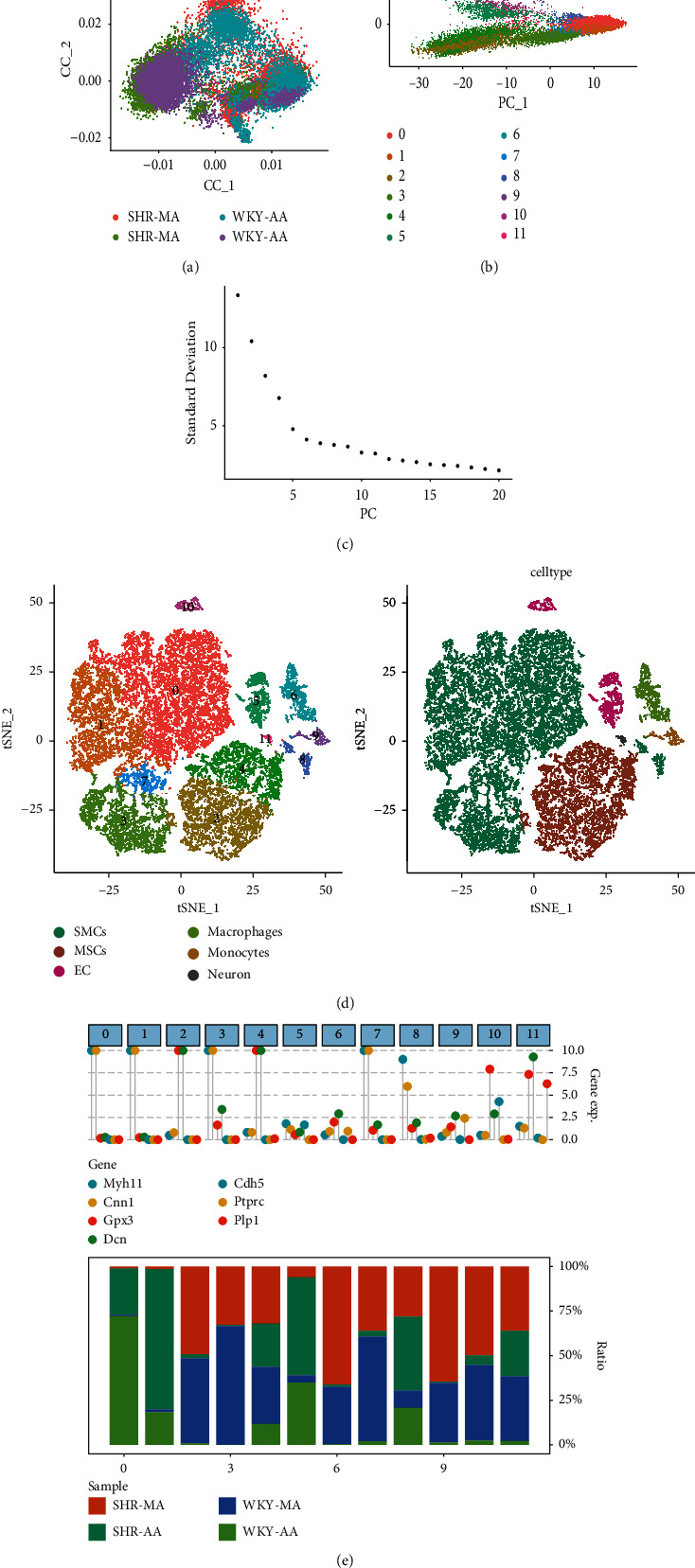
Heterogeneity of arterial vascular cells. Distribution status of each sample and cluster after CCA integration analysis (a, b). Twenty PCs identified via PCA with an estimated *p* < 0.05 (c). Clustering of mouse arterial cells and tSNE typing of each cell subpopulation (d). Enrichment of tissue samples in each cluster and expression of marker genes in different subgroups (e).

We analyzed 11798 cells from the SHR group and 12589 cells from the WKY group of mouse arterial cells, and the RNA expression profile is shown in Supplementary Figure 1A. The distribution states of each sample and cluster after the canonical correlation analysis (CCA) integration analysis to remove the batch effect are shown in Figures 1(a) and 1(b). Then, PCA identified 20 PCs with an estimated *p* value < 0.05 (Figure 1(c)). A tSNE downscaling analysis was performed using the top 11 principal components, and the tSNE localization of single cells and key molecules was visually mapped for all samples (Figure 1(d)). Based on the study by Cheng et al., these cells were clustered into six cell types: SMCs, MSCs, EC, macrophages, monocytes, monocytes, and neurons [18] (Figure 1(d)). The enrichment of tissue samples in each cluster and the expression of marker genes in the different subgroups are shown in Figure 1(e). The distribution characteristics of the cells in the SHR and WKY are shown in Supplementary Figure 1B. The enrichment of tissue samples in each cluster and the expression of marker genes in different subgroups are also shown (Figure 1(e) and Supplementary Figure 1C).

### 3.2. Neuron-Related Cellular Communication

**Figure 2:**
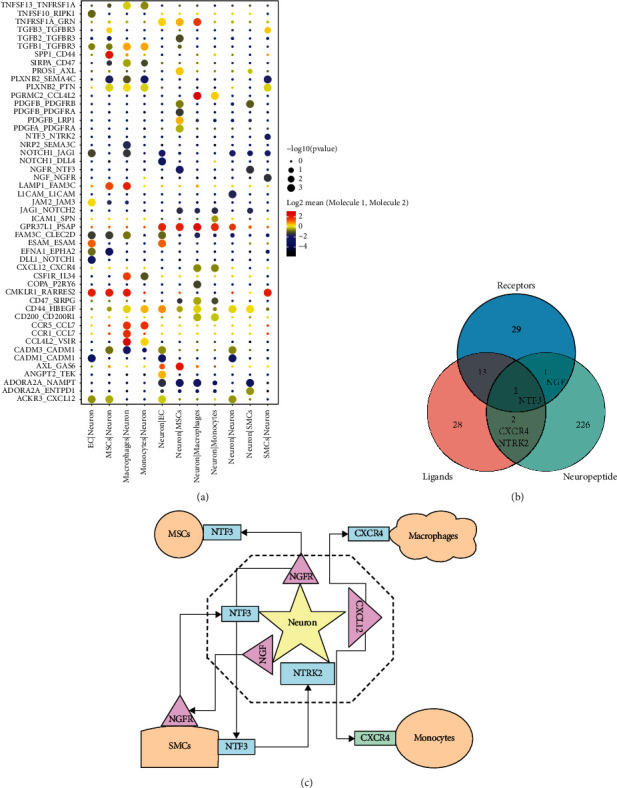
Analysis of cellular communication between different cell subsets. Analysis of cellular communication between neuronal cells and other cell subpopulations (a). Venn diagram analysis suggesting that Ngf, Ntf3, Cxcr4, and Ntrk2 are key neuropeptide-related genes involved in the communication between vascular smooth muscle cells and neuronal cells (b). Schematic representation of the interactions between key neuropeptides and their ligands or receptors in vascular cell communication (c).

CellPhoneDB was used for cell communication analysis between neuronal cells and other cell subpopulations (Figure 2(a)). These key ligand-receptor interaction genes were extracted for Venn diagram analysis (Figure 2(b)). The results suggested that Ngf, Ntf3, Cxcr4, and Ntrk2 were key neuropeptide-related genes involved in the communication between vascular smooth muscle cells and neurons. A schematic representation of the interactions between these key neuropeptides and their ligands or receptors in vascular cell communication is shown in Figure 2(c).

### 3.3. Creb3l1 Was Found to Be a Key Transcription Factor Regulating Ntrk2 in Neuron

**Figure 3:**
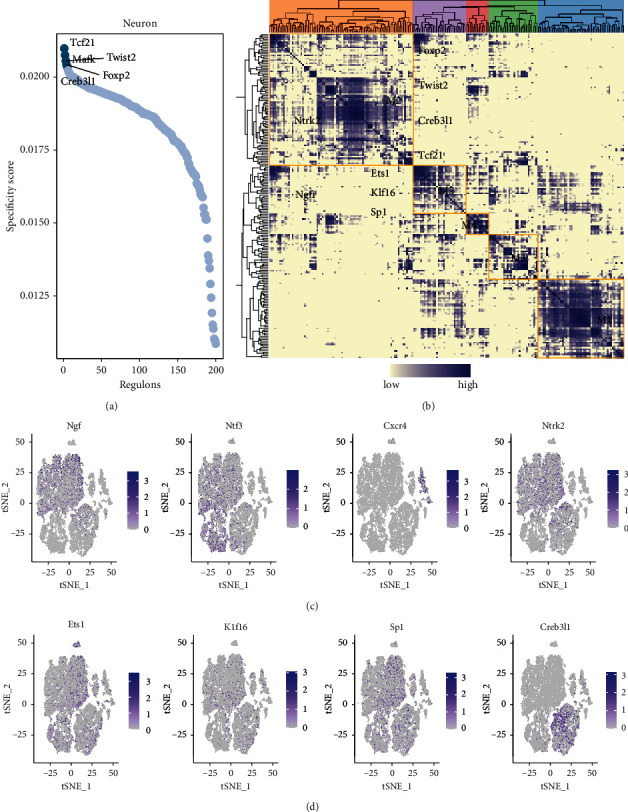
Definition and identification of key transcription factors. Analysis of neuron-specific transcription factor activity (a). Cluster map of identified key transcription factors with Foxp2, Twist2, Creb3l1, and Tcf21 located in the M2 module while Ets, Klf16, and Sp1 are located in the M5 module (b). Distribution of tSNE of identified core transcription factors (Ngf, Ntf3, Cxcr4, and Ntrk2) and their expression levels in each subpopulation of cells (c). Distribution of tSNE of key neuropeptide-related genes Ets1, Klf16, Sp1, and Creb3l1 and their expression in various subpopulations of cells (d).

The SCENIC method was used to analyze key transcription factors in neurons. The top five transcription factors were identified as Tcf21, Mafk, Twist2, Foxp2, and Creb3l1 (Figure 3(a)).

All the identified key transcription factors were clustered into different modules. Foxp2, Twist2, Creb3l1, and Tcf21 were found to be located in the M2 module, with Ntrk2 possibly being regulated by these TFs. Meanwhile, Ets, Klf16, and Sp1 were located in the M5 module and possibly regulated NTF3 (Figure 3(b)). It should be noted that M2 and M5 refer to cell clustering based on transcription factors rather than specific cell types. The tSNE distribution of Ngf, Ntf3, Cxcr4, and Ntrk2 and their expression in each subpopulation of cells are shown in Figure 3(c). Ntrk2 was found to be enriched in neurons, while Cxcr4 was found to be enriched in macrophages. Meanwhile, Ngf, Ntf3, and Ntrk2 were all found to be enriched in SMCs.

Finally, the tSNE distribution of Ets1, Klf16, Sp1, and Creb3l1—the main transcription factors regulating Ntrk2 and NTF3—and their expression in each subpopulation of cells were shown in Figure 3(d). Creb3l1 was found to be highly expressed in MSCs and neurons. Thus, Creb3l1 may act as a key transcription factor in neuronal cells upregulating Ntrk2.

### 3.4. Ntrk2 Upregulation Was Found to Be Associated with Ferroptosis-Related Pathways

**Figure 4:**
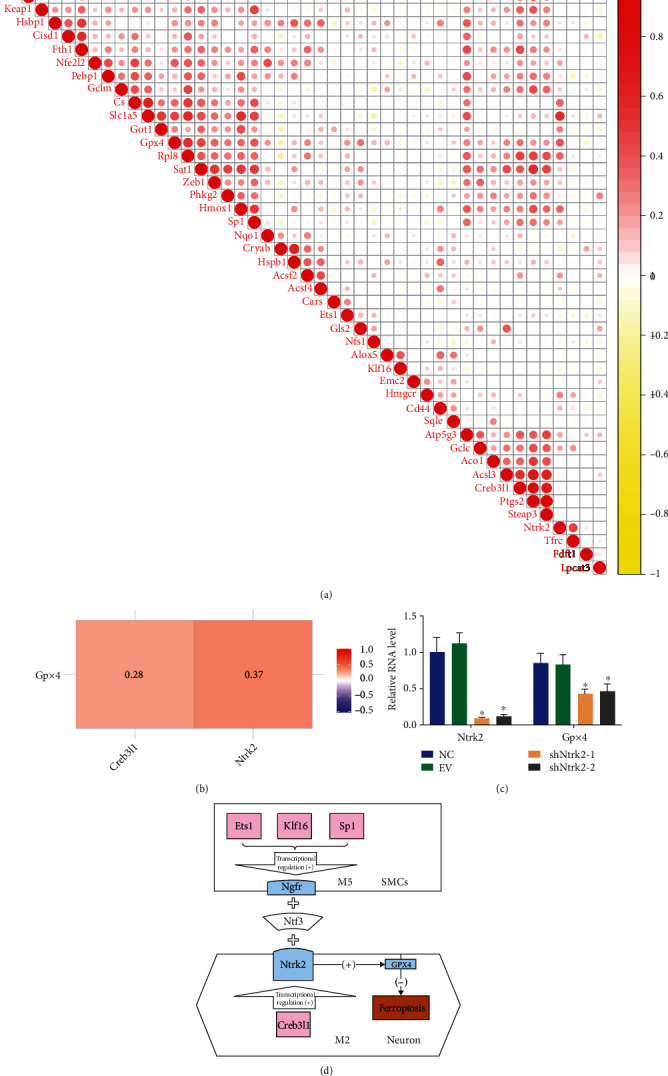
Ntrk2 upregulation was found to be associated with ferroptosis-related pathways. Correlation analysis of Ntrk2 and ferroptosis-related genes. Ntrk2 expression was found to be significantly correlated with Gpx4 (a). In vascular neurons, Gpx4 expression was found to be positively correlated with Creb3l1 and Ntrk2 (b). RT-qPCR graphs showing the association between Ntrk2 and Gpx4 (c). The potential mechanism of upregulation of Ntrk2 in a hypertensive microenvironment to induce ferroptosis (d).

Ferroptosis is an iron-dependent form of programmed cell death distinct from apoptosis, cell necrosis, and autophagy. Ferroptosis is marked by a decrease in the levels of glutathione peroxidase 4 (Gpx4), the regulatory core enzyme of the antioxidant or glutathione system [28]. We have tried to explore the association of Creb3l1 and Ntrk2 with ferroptosis. Correlation analysis of Ntrk2 and ferroptosis-related genes suggested that Ntrk2 expression was found to be significantly associated with Gpx4 (Figure 4(a)). In vascular neurons, Gpx4 expression was found to be positively correlated with Creb3l1 and Ntrk2 (Figure 4(b)). The RT-qPCR experiments indicate that after Ntrk2 expression was downregulated in HT22 cells, the expression of Gpx4 was also downregulated (Figure 4(c)). We thus come up with a hypothesis that Creb3l1, a key transcription factor in neurons, may upregulate Ntrk2 in the hypertensive microenvironment to promote vascular smooth muscle cell-neuron interactions and thereby potentially prevent ferroptosis in neurons (Figure 4(d)).

## 4. Discussion

This study identifies by SCENIC that Creb3l1 may upregulate Ntrk2 to promote vascular smooth muscle cell-neuron interaction and thereby potentially prevent ferroptosis in neurons.

SCENIC is a method to compute single-cell transcriptome data for gene regulatory network reconstruction and cell state identification based on gene regulatory network and motif analysis. CREB3L1 (cAMP-responsive element-binding protein 3-like 1) is a transcriptional promoter of AVP expression under the regulation of cAMP [29]. This suggests that CREB3L1 may play an important role in blood pressure regulatory functions. However, variations on the expression of CREB3L1 in hypertensive patients remain to be investigated. Under the positive regulation of cAMP, Creb3l1 activates the Avp promoter, which increases AVP expression.

The results of the CellPhoneDB ligand-receptor analysis suggested that Ntrk2 was a key neuropeptide-related gene involved in the communication between vascular smooth muscle cells and neurons. Ntrk2 was found to be enriched in neurons in this study. And Ntrk2 (neurotrophic receptor tyrosine kinase 2), also known as tyrosine kinase receptor B (TrkB), and its ligands are novel targets for angiogenic therapy [30, 31]. It is a cell surface receptor that can be activated through ligand binding and is expressed in endothelial cells and the smooth muscle cells of the developing and adult heart [32, 33]. Ntrk2 is aberrantly expressed in endothelial cells under inflammatory conditions [34]. BDNF is the preferred ligand for Ntrk2 in the neuronal environment [35]. However, it is not the only neurotrophic factor that can activate Ntrk2. Ntf3 (neurotrophin-3) can also bind and activate Ntrk2, which was also found in this study [36].

Our results suggested that Ntrk2 expression was significantly correlated with Gpx4. RT-qPCR experiments confirmed that when Ntrk2 expression was downregulated in HT22 cells, Gpx4 expression was also downregulated. In the absence of intracellular glutathione, the activity of the key regulatory enzyme Gpx4 is reduced and the reduction reaction it catalyzes becomes unable to metabolize lipid peroxides. Gpx4, like other selenium-containing Gpx enzymes, is an antioxidant. Its catalytic center is a tetramer consisting of a hydrogen bond between the Sec residues and nitrogen atoms of asparagine, glutamine, and tryptophan. Loss of Gpx4 expression or activity through genetic or pharmacological pathways thus promotes ferroptosis in a lipid ROS-dependent manner [37]. Recent genetic and epidemiological studies showed that reduced Gpx4 levels or reduced catalytic activity led to obesity, cardiovascular disease, and inflammation [38–40]. Decreased levels of Gpx4 can cause increased lipid peroxidation, resulting in ferroptosis in vascular endothelial cells, which promotes thrombosis and the development of atherosclerosis. Overall, Gpx4 prevents cellular ferroptosis by eliminating intracellular lipid ROS, while inhibition of Gpx4 induces ferroptosis [41]. The binding of Ntf3 to Ntrk2 increased Gpx4 levels, attenuated fat oxidation, inhibited ferroptosis, and protected the vascular smooth muscle cells and neurons.

Creb3l1, a key neuronal transcription factor in a hypertensive microenvironment, may upregulated Ntrk2 expression. Creb3l1 has been mostly found to be associated with tumor progression in the nervous system [42]. In other studies, the homeostasis of Creb3l1 has also been found to play an important role in maintaining the function of vascular integrity [43]. Our study suggests a potential function of Creb3l1 in the homeostasis of the hypertensive microenvironment.

Despite these promising results, there is a need to further verify the potential regulatory role of Crebl1 on Ntrk2 and Ntf3. This study is based on single-cell sequencing data obtained from a single dataset, which has its own limitations. The data of this study were obtained from a single dataset. There is a need to integrate multiomics technologies such as single-cell proteomics, single-cell immunomics, and spatial transcriptomics to better understand the complex interactions between AS initiation, progression, regression, and plaque rupture. As the cost of single-cell sequencing gradually decreases, correlations between single-cell sequencing data and clinical parameters may be established in the future to translate into clinical applications and eventually develop new therapeutic strategies for controlling hypertension.

## 5. Conclusions

We showed that Creb3l1, a key transcription factor in neurons, upregulates Ntrk2 expression in the hypertensive microenvironment. The binding of Ntf3 to Ntrk2 increases Gpx4 levels, inhibits ferroptosis, and protects vascular smooth muscle cells and neurons.

## Data Availability

Single cells of arterial vascular cells were obtained from the GSE149777 dataset of the GEO database.

## Abbreviations

ARE: Antioxidant response elements
CREB3L1: Camp-responsive element-binding protein 3-like 1
DBP: Diastolic blood pressure
GRN: Gene regulatory network
Gpx4: Glutathione peroxidase 4
KY Rats: Healthy Controls Wistar Rats
Hvg: High-variable gene
Ntrk2: Neurotrophic receptor tyrosine kinase 2
NTF3: Neurotrophin-3
PTS: Prethrombotic state
PCA: Principal component analysis
ROS: Reactive oxygen species
SCENIC: Single-cell regulatory network inference and clustering
SHR: Spontaneously hypertensive rats
SBP: Systolic blood pressure
Trkb: Tyrosine kinase receptor B.

## Supplementary Materials

Supplementary Materials All supplementary documents were included in the document “sub Figures.doc”.
