# Supplementary material for: The Neuronal Transcription Factor Creb3l1 Potential Upregulates Ntrk2 in the Hypertensive Microenvironment to Promote Vascular Smooth Muscle Cell-Neuron Interaction and Prevent Neurons from Ferroptosis: A Bioinformatic Research of scRNA-seq Data
Source: Dis Markers. 2022 Feb 10;2022:8339759. doi: 10.1155/2022/8339759 (PMC8853773; doi:10.1155/2022/8339759)
Supplement: Supplementary Materials — All supplementary documents were included in the document “sub Figures.doc”. [file 8339759.f1.docx]

# Supplementary Figure Legends


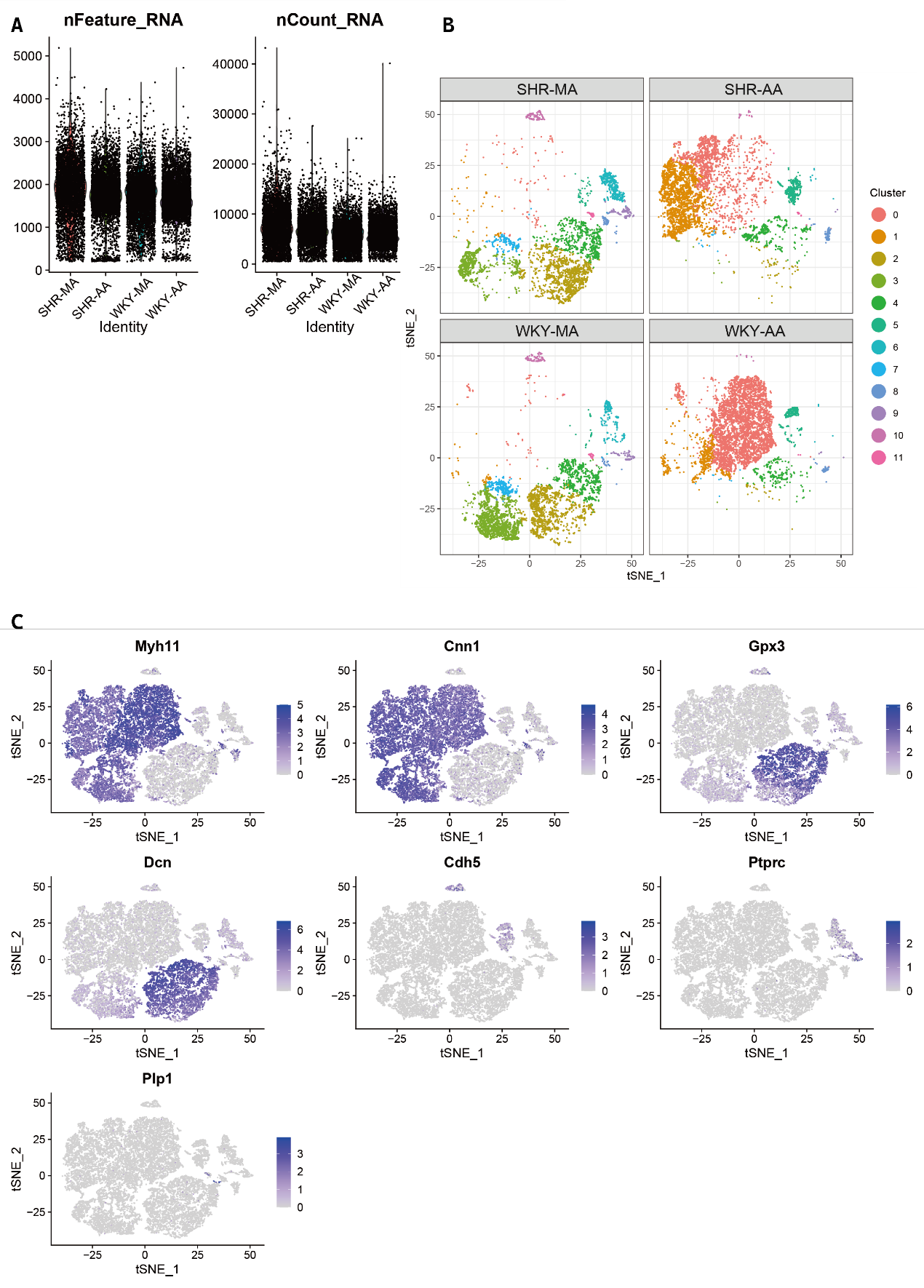


**Supplementary figure 1. Characterization of vascular arterial cells.** RNA expression characteristics of 11798 cells from the SHR group and 12589 cells from the WKY group (A)；Distribution characteristics of cells in the SHR and WKY groups in the tSNE plot (B)；tSNE distribution of key marker genes (Myh11, Cnn1, Gpx3, Dcn, Cdh5, Ptprc and Plp1) and their expression in various subpopulations of cells (C).
